# Association Between Ankle‐Brachial Index and Risk of Early Neurological Deterioration After Acute Isolated Pontine Infarction

**DOI:** 10.1002/brb3.70481

**Published:** 2025-04-09

**Authors:** Dong‐Bo Liu, Bing‐Xian Zhang, Yao Zhou, Jian‐Hua Zhao, Jie‐Wen Zhang

**Affiliations:** ^1^ Department of Neurology Henan Provincial People's Hospital (People's Hospital of Zhengzhou University) Henan Provincial People's Hospital (Zhengzhou University People's Hospital), No.7 Weiwu Road Zhengzhou Henan Province China

**Keywords:** ankle‐brachial index, neurologic deterioration, neuroprotective effect, predictor, pontine infarction

## Abstract

**Introduction:**

Lower ankle‐brachial index (ABI) has been linked to worse prognosis after ischemic stroke, including greater risk of recurrence. Whether the index also correlates with risk of early neurological deterioration after acute cerebral infarction is unclear.

**Material and methods:**

We prospectively analyzed patients admitted to our hospital between February 2019 and January 2024 for acute isolated pontine infarction and who showed an ABI no greater than 1.40. Patients were classified into three groups according to whether their index was low (≤0.90), borderline (0.91–1.10), or normal (1.11–1.40). We compared the index between patients who showed early neurological deterioration or not, which was defined as an increase of at least one point on the motor item on the National Institute of Health Stroke Scale (NIHSS) or at least two points on the total NIHSS score during the first week after admission.

**Results:**

Of the 408 patients in our analysis, 120 (29.4%) showed early neurological deterioration. ABI showed the following distribution: low, 108 patients (26.5%); borderline, 136 (33.3%); and normal, 164 (40.2%). In multivariate regression, borderline values of ABI (0.91–1.10) were associated with significantly lower risk of early neurological deterioration (OR 0.457, 95% CI: 0.246–0.848), whereas low values (≤0.90) were associated with significantly higher risk (OR 1.975, 95% CI: 1.110–3.512).

**Conclusions:**

Low ABI may independently predict increased risk of early neurological deterioration following acute isolated pontine infarction. Borderline ABI, in contrast, may independently predict reduced risk.

## Graphical abstract

The ankle‐brachial index (ABI) is the ratio of the systolic blood pressure at the ankle to that at the brachial artery, with a normal range of 0.91–1.00. Our study revealed that a low ABI (≤0.90) may independently predict an increased risk of early neurological deterioration subsequent to acute isolated pontine infarction. In contrast, a borderline ABI (ranging from 0.91 to 1.10) may independently predict a decreased risk.

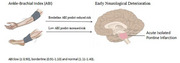

## 1 Introduction

Acute isolated pontine infarction (AIPI) accounts for 7%–15% of acute cerebral infarctions (Du J et al. 2018), and many affected individuals suffer early neurological deterioration (END), which is associated with more serious morbidity and higher risk of mortality (Li et al. 2020; Seners et al. 2015). Identifying clinicodemographic factors that predict such deterioration may help screen for patients at higher risk and thereby facilitate more intensive management and earlier intervention.

We wondered whether one such predictor might be the ankle‐brachial index (ABI), which is the ratio of systolic blood pressure at the ankle to that at the brachial artery. This index has already proven useful as a non‐invasive criterion when diagnosing peripheral artery disease in the legs (Aboyans et al. 2012;Gerhard Herman et al. 2017;Horie 2024), and numerous studies have linked lower values of the index to greater risk of worse prognosis after ischemic stroke, including higher risk of recurrence (Abboud et al. 2019; Han et al. 2021, 2022). Here, we explored whether the index might be useful for identifying patients at higher risk of END after AIPI.

## 2 Methods

### 2.1 Patients

We prospectively analyzed a consecutive series of patients who were admitted to the Department of Neurology at Henan Provincial People's Hospital between February 2019 and January 2024 and who (1) were diagnosed with acute ischemic stroke according to national guidelines (Chinese Medical Association 2018), (2) were admitted to our hospital within 48 h of stroke onset, (3) were examined by magnetic resonance imaging (MRI) to confirm infarction only in the pons (see below), and (4) were at least 18 years old. We excluded patients (1) who received thrombolysis or thrombectomy treatment after onset, (2) who were pregnant or had other diseases that prevented complete neurological examination, (3) whose condition worsened due to respiratory or heart failure or infection within 1 week after admission, or (4) for whom the ABI was > 1.40 or unavailable.

This study was approved by the Ethics Committee of Henan Provincial People's Hospital ([2018] Lunshen No. 38]) Patients or their legal guardians provided written informed consent at enrollment.

### 2.2 Data Collection and Calculation of the ABI

Medical and treatment histories were collected during a standardized interview involving the patient and family members. Data were collected on age, sex, risk factors for stroke (hypertension, diabetes, hypercholesterolemia, coronary artery disease, smoking, atrial fibrillation), previous antiplatelet or lipid‐lowering therapy, and previous stroke or transient ischemic attack.

The vascular risk factors assessed at baseline (Li et al. 2013) included hypertension, defined as blood pressure ≥140/90 mmHg or current antihypertensive therapy; diabetes, defined as fasting blood glucose ≥7.0 mmol/L, blood glucose ≥11.1 mmol/L at 2 h after a meal, or current hypoglycemic therapy; and hypercholesterolemia, defined as total cholesterol ≥5.17 mmol/L or current lipid‐lowering drugs. Coronary artery disease included stable or unstable angina, myocardial infarction, coronary artery surgery, or angioplasty. Atrial fibrillation was diagnosed based on at least one electrocardiogram prior to or during hospitalization (Tsivgoulis et al. 2012). Prior stroke did not include lacunar infarction. Prior transient ischemic attack was defined as transient focal neurological dysfunction due to ischemia affecting the brain, spinal cord, or retina, without permanent tissue damage due to transient ischemia.

During hospitalization, the ABI was measured while patients were supine using a previously validated automatic blood pressure monitor (Omron, Japan). The ABI was calculated as the ratio of ankle systolic blood pressure to the systolic blood pressure of the upper arm on the side with the higher value. After obtaining ABI values for both sides, the lower ABI value was used for analysis (Matsushita et al. 2017). Patients were classified into three groups according to whether their index was low (≤0.90), borderline (0.91–1.10), or normal (1.11–1.40).

### 2.3 Diagnosis of Early Neurological Deterioration

At admission as well as on days 3, 5, and 7, patients were assessed for neurological symptoms using the National Institutes of Health Stroke Scale (NIHSS). Patients were diagnosed with early neurological deterioration if, during the first week after admission, their score on the motor item increased by at least one point or their total NIHSS score increased by at least two points (Kwon et al. 2014).

Within 48 h after admission, all patients were analyzed by MRI of the head on a 3.0‐T Trio Tim system (Siemens, Germany). Imaging was repeated if a patient's clinical condition changed substantially. Isolated pontine infarction was defined as the presence of focal lesions only in the pons, based on diffusion‐weighted imaging. The degree of basilar artery stenosis was determined independently by two experienced radiologists from three‐dimensional time‐of‐flight magnetic resonance angiograms. Disagreements between the two radiologists were resolved through discussion.

### 2.4 Statistical Analysis

All data were analyzed using SPSS 21.0 for Windows (IBM, Armonk, NY, USA). Continuous data were confirmed to be normally distributed using the Kolmogorov–Smirnov test. Intergroup differences in continuous data were assessed for significance using Student's *t*‐test or the Mann–Whitney *U* test. Differences in categorical data were assessed using the chi‐squared or Fisher's exact tests.

Clinicodemographic factors that were associated with early neurological deterioration in univariate regression with *p* < 0.1 were entered into multivariable logistic regression in order to identify independent risk factors.

## 3 Results

### 3.1 General Characteristics of Study Population

During the study period, 462 patients met the inclusion criteria, but 54 were excluded because they had received thrombolysis or thrombectomy after infarction (*n* = 27), their ABI exceeded 1.40 or was unavailable (*n* = 12), they did not complete neurological exams (*n* = 6), their condition worsened due to other diseases (*n* = 6), or they were followed up for less than 1 week (*n* = 3). The 408 patients in the final analysis (62.0% men) were a mean of 65.73 ± 10.33 years old and showed the following distribution of ABI: low, 108 (26.5%); borderline, 136 (33.3%); and normal, 164 (40.2%).

### 3.2 Various Clinical Manifestations of Study Population

Among the 408 patients studied, 120 patients (29.4%) demonstrated END, 302 (74.0%) exhibited unilateral limb weakness, 224 (54.9%) dysphasia, 144 (35.3%) rotational visual disturbances, 118 (28.9%) unilateral hyposensation, 60 (14.7%) ataxia, 32 (7.8%) diplopia, and 21 (5.1%) cerebellar dysarthria. Among the 89 patients presenting with clinical lacunar syndromes (with or without incomplete deficit), 73 (82.0%) were attributed to lacunar infarcts. The distribution of these syndromes showed the following frequencies: pure motor hemiparesis was observed in 36 patients (40.4%), pure sensory syndrome in eight (9.0%), sensorimotor syndrome in 33 (37.1%), ataxic‐hemiparesis in three (3.4%), dysarthria‐clumsy hand syndrome in two (2.2%), and atypical lacunar syndromes in seven patients (7.9%).

### 3.3 Predictors and Protective Factors of END: Univariate and Multivariable Analysis

Univariate analysis linked the following characteristics to such deterioration (*p* < 0.05; Table 1): male sex, age, NIHSS score at admission, infarction location in the lower pons, basilar artery stenosis ≥50%, and borderline or low ABI. The seven variables mentioned above, along with diabetes mellitus (*p*< 0.1), were included in the multivariable model, from which four variables emerged as significant independent predictors of deterioration (*p* < 0.05): age, NIHSS score at admission, infarction location in the lower pons and basilar artery stenosis ≥50%. A multivariable model that accounted for these four variables identified borderline ABI as a protective factor against early neurological deterioration (OR 0.457, 95% CI: 0.246–0.848) and low ABI as a predictor of such deterioration (OR 1.975, 95% CI: 1.110–3.512; Table 2).

**TABLE 1:** Comparison of clinicodemographic characteristics between patients who experienced early neurologic deterioration or not after acute isolated pontine infarction.

Characteristic	Deterioration (*n* = 120)	No deterioration (*n* = 288)	*p*
Men	65(54.2)	188(65.3)	0.035
Age (year)	69.27 ± 9.48	64.26 ± 10.32	< 0.001
Risk factors for stroke			
Hypertension	89 (74.2)	198 (68.8)	0.275
Diabetes	63 (52.5)	123 (42.7)	0.070
Hypercholesterolemia	52(43.3)	110(38.2)	0.334
Coronary artery disease	16(13.3)	32(11.1)	0.526
Current smoking	32(26.7)	58(20.1)	0.147
Atrial fibrillation	7 (5.8)	10 (3.5)	0.277
Previous stroke or transient ischemic attack	49 (40.8)	124 (43.1)	0.679
Infarct location in pontine region			
Upper	16(13.3)	56(20.0)	0.112
Middle	37(30.8)	109(37.8)	0.178
Lower	47(39.2)	83(28.8)	0.041
Multiple	20(16.7)	40(13.9)	0.470
Basilar artery stenosis ≥50%	51(42.5)	65(22.6)	< 0.001
History of anti‐platelet therapy	49(40.8)	107(37.2)	0.486
History of anti‐lipidic therapy	38(31.7)	81(28.1)	0.473
NIHSS score at admission	5 [4–7]	4 [3–5]	< 0.001
Category of ankle‐brachial index			
Normal (1.11–1.40)	46(38.3)	118(41.0)	_
Borderline (0.91–1.10)	24(20.0)	112(38.9)	< 0.001
Low (≤0.90)	50(41.7)	58(20.1)	< 0.001

*Note*: Values are *n* (%), mean ± SD or median [interquartile range], unless otherwise noted.

Abbreviation: NIHSS, National Institutes of Health Stroke Scale.

**TABLE 2:** Multivariate logistic regression to identify independent predictors of early neurological deterioration after acute isolated pontine infarction.

Variable	*β*	Odds ratio (95% confidence interval)	*p*
Male sex	−0.316	0.729(0.442–1.203)	0.216
Age	0.051	1.052(1.026–1.078)	< 0.001
Diabetes	0.405	1.500(0.913–2.462)	0.109
NIHSS score at admission	0.363	1.438(1.258–1.643)	< 0.001
Infarct location in lower pons	0.576	1.878(1.126–3.134)	0.031
Basilar artery stenosis ≥50%	0.718	2.050(1.202–3.496)	0.008
Category of ankle‐brachial index			
Normal (1.11–1.40)		Reference	
Borderline (0.91–1.10)	−0.782	0.457(0.246–0.848)	0.013
Low (≤0.90)	0.680	1.975(1.110–3.512)	0.021

Abbreviation: NIHSS, National Institute of Health Stroke Scale.

## 4 Discussion

Our single‐center prospective study provides evidence that borderline values of the ABI (0.91–1.10) are associated with significantly lower risk of END after AIPI, while low values (≤0.90) are associated with significantly higher risk. These insights may aid in identifying individuals at higher risk of END after AIPI, which may guide more intensive management and targeted interventions.

Our data indicate that although lacunar syndrome highly suggests small pontine cerebral infarction, 18.0% of lacunar syndrome cases are not caused by lacunar cerebral infarction, which is generally consistent with the findings of an earlier study (Arboix et al. 2010). This discovery not only underscores the diversity of etiologies underlying lacunar syndrome but also highlights the necessity of assessments such as brain MRI in differentiating the causes of cerebral infarction.

The prevalence of low ABI in our sample of patients who suffered AIPI (26.5%) falls within the range of 24%–51% reported for patients of acute ischemic stroke (Agnelli et al. 2006; Busch et al. 2009; Purroy et al. 2010; Weimar C et al. 2008). We found a prevalence of 33.3% for borderline ABI, which we cannot compare to the literature for lack of relevant studies among patients of acute cerebral infarction. Future studies should examine this prevalence, especially since our data suggest that it protects against END.

Our observation of an association between low ABI and greater risk of deterioration is consistent with its known association with arterial stenosis in lower extremities (Horie 2024; Lee et al. 2022; Polonsky and McDermott et al. 2021), which is a particularly useful diagnostic marker given that up to 70% of such cases of arterial stenosis are asymptomatic (Busch et al. 2009). Our observation is also consistent with the known association of low ABI with poor outcomes after ischemic stroke (Abboud et al. 2019; Han et al. 2022), potentially including higher risk of stroke recurrence and mortality due to vascular events (Hong et al. 2016). One possibility is that low ABI indicates stenosis in large intra‐ and extracranial arteries (Chang et al. 2023), which may increase risk of END after stroke. Future studies should explore the pathology underlying the observed association between low index and higher risk of deterioration.

Our observation of an association between borderline ABI and lower risk of END is, to our knowledge, the first exploration of relationships between borderline values and stroke. Potentially consistent with our results, a trial of ischemic stroke patients linked the presence of peripheral artery disease that did not require revascularization and was therefore presumably relatively mild (Connolly et al. 2013). Although that study did not report ABI, we presume that those patients with peripheral artery disease had indices only slightly lower than normal. Potentially in contrast to our results, studies of the general population showed risk of cardiovascular disease to be slightly higher among those with borderline values of the ABI than among those with normal values (Ankle Brachial Index Collaboration et al. 2008). Future studies should verify the neuroprotective effect of borderline ABI after AIPI and seek to explain the mechanisms involved, which may prove useful as treatments.

For example, exposing the limbs to brief cycles of ischemia and reperfusion (remote ischemic conditioning) has shown promise for improving the prognosis of individuals with cerebrovascular disease (Zhao et al. 2023). The chronic hypoperfusion in lower limbs in peripheral artery disease may work through a similar mechanism to exert neuroprotective effects (Connolly et al. 2013). We speculate that chronic limb ischemia can exert neuroprotective effects when the ABI lies within the borderline range 0.91–1.10, while the harmful effects of ischemia outweigh any benefits at index values ≤0.90. Suitably large studies should explore the optimal ABI for maximizing neuroprotective effects while minimizing harmful ones.

Drawing upon the established research paradigm (Abboud et al. 2019), we encountered a challenge in the form of limited data, particularly the rarity of patient samples exhibiting ABI values surpassing 1.40, which hindered our capacity to delve into a comprehensive analysis of this specific subgroup. Moreover, to uphold the internal coherence and precision of our research outcomes, we devised a judicious sample selection methodology, excluding those who underwent thrombolytic therapy or thrombectomy, as these interventions hold the potential to significantly alter the course and eventual outcome of AIPI (Qingke et al. 2021; Thomalla et al. 2018). While this strategy effectively safeguarded the homogeneity of our study cohort, it is imperative to acknowledge that it may have inadvertently narrowed the representativeness of our sample in comparison to the broader AIPI patient population.

Our findings should be interpreted with caution given that our sample was relatively small and came from a single center. We cannot exclude that we missed cases of END that occurred prior to admission. None of our patients was admitted to the intensive care unit, so the generalizability of our results to such patients is unclear. Future studies, preferably from multiple centers, should verify and extend our results.

## 5 Conclusion

Despite these limitations, our study provides the first evidence that lower ABI is associated with higher risk of END after AIPI, whereas borderline index is associated with lower risk, perhaps because chronic hypoperfusion in lower extremities protects against such deterioration. Our findings should be further explored in order to develop ways to screen for stroke patients at higher risk of END and develop treatments to mitigate or even prevent it.

## Author Contributions

**Dong‐Bo Liu**: Methodology; investigation; writing–original draft; conceptualization. **Bing‐Xian Zhang**: Software; data curation; investigation; validation. **Yao Zhou**: Methodology; software; data curation; supervision; formal analysis. **Jian‐Hua Zhao**: Conceptualization; funding acquisition; resources; supervision. **Jie‐Wen Zhang**: Conceptualization; methodology; resources; supervision; project administration; funding acquisition; writing–review and editing.

## Conflicts of Interest

The authors declare no conflicts of interest.

### Peer Review

The peer review history for this article is available at https://publons.com/publon/10.1002/brb3.70481

## Data Availability

The data that support the findings of this study are available from the corresponding author upon reasonable request.
